# News and Coming Events

**Published:** 1908-05-30

**Authors:** 


### News and Coming Events.

Sir Isambard Owen has been re-elected Senior Deputy-Chancellor of the University of Wales.

The Third International Congress for the Care of the Insane will be held in Vienna from October 7 to 11.

Lieut.-Col. and Brevet Col. David Bruce, C.B., M.B., R.A.M.C., has been promoted to the rank of Colonel in the Army Medical Service.

The Earl of Cromer will preside at the inaugural meeting of the Research Defence Society to be held at 20 Hanover Square, on Friday, June 19, at 5 o'clock.

A. P. Parker, M.A., B.M., F.R.C.S.Eng., of Magdalen College, has been appointed University Lecturer in Applied Anatomy in the University of Oxford.

Herbert John Allcroft, Esq., F.R.G.S., treasurer of the Royal Hospital for Incurables, Putney Heath, will preside at the annual dinner, which will be held at the Savoy Hotel, on Thursday, June 18, at 6.30 for 7 p.m.

The Committee for the Removal of King's College Hospital to South London has received a cheque for £500, being the first instalment of a promised donation of £2,500, from the Ecclesiastical Commission in aid of the fund.

At a general meeting of the Medical Society of London, held at 11 Chandos Street, Cavendish Square, London, Mr. C. B. Lockwood was elected President for the ensuing year.

The Hospital for Consumption and Diseases of the Chest, Brompton, has received a donation of £100 from the Council of the City of London Corporation in response to its urgent appeal for funds.

The Derbyshire Infirmary announces that the sum of £5,000 has been received from Mr. A. Leslie Wright, J.P., of Butterley Hall, and a further sum of from £2,000 to £3,000 has been promised by him to build and equip children's wards in memory of his late wife.

At the last Comitia of the Royal College of Physicians of London the President proposed, and Dr. Theodore Williams seconded, that a conversazione should be given in the College in the course of the summer and that a committee should be appointed to make the necessary arrangements. The proposal was carried.

At the 25th annual meeting of the Paddington Green Children's Hospital, Mr. Douglas Owen, who presided, said that during the past year the executors of the late Mr. Samuel Lewis had forwarded £4,250 in part payment of the legacy of £5,000 bequeathed for the founding and endowing of a ward. Efforts were being made to improve the hospital accommodation, and towards that object additional funds were urgently needed.

Lord William Cecil presided at the annual meeting of the Queen's Hospital for Children, held on May 21, at the Institution, Hackney Road. The Chairman said the past year had been a satisfactory one in every way, and he hoped the kindness of her Majesty in permitting the hospital to bear her name would lead to renewed effort. He hoped the bazaar which her Royal Highness Princess Henry of Battenberg—who took a great interest in the hospital—would open on June 23 at Shoreditch Town Hall, would be liberally supported by the public, so that the Committee could clear off their debt of £8,000.

We have received notices of the following donations fro[illegible] the Trustees of Smith's (Kensington Estate) Charity: Th[illegible] Chelsea Hospital for Women, £250; the Royal Dental Ho[illegible] pital, £250; the Royal National Orthopædic Hospital £250; and Queen Charlotte's Lying-in Hospital, £123.

The Countess of Bective, who moved the adoption o[illegible] the accounts at the annual meeting of the Guy's Hospital Ladies' Association, held at the hospital, mentioned that the Association had this year been able to make a donation of £300 to support six beds in the maternity ward.

Viscount Morley of Blackburn, O.M., H.M. Secretary of State for India, will be the guest of the evening at the annual dinner of the officers of the Indian Medical Service at the Gaiety Restaurant, Strand, London, on Thursday, June 18, at 7.45 p.m. The honorary secretary is Lieut.-Col. P. J. Freyer, 27 Harley Street, W.

At the London Hospital Medical College a course of four lectures will be delivered by Mr. Jonathan Hutchinson, F.R.S., F.R.C.S., Emeritus Professor of Clinical Surgery to the hospital, on Tuesdays at 3 p.m. The subjects of the lectures are as follows: June 16—The Liver and Jaundice in Reference to Skin Diseases; June 23—Stigmata Insectorum; June 30—Vegetable Pathology; and July 7—The Present State of the Leprosy Question. The lectures are free to all students of the University of London and to medical graduates.

At University College, London, elections will take place in September to the following Medical Entrance Scholarship and Exhibitions: Bucknill Scholarship, value 135 guineas, entitling the holder to the Intermediate Medical Course (including Part II. of the Preliminary Scientific) at University College, and the Final M.B., B.S. at the University College Hospital Medical School. Two exhibitions of the value of 55 guineas each entitling the holder to the Intermediate Medical Course (including Part II. of the Preliminary Scientific) at University College.

The annual provincial meeting of the British Balneological and Climatological Society was held at Harrogate under the presidency of Dr. W. J. Tyson on Saturday and Sunday, May 16 and 17. About 135 Fellows and guests attended this meeting, which proved most instructive, enjoyable, and successful. These provincial meetings are very popular with the Fellows of the Society, and have become an established feature. The proceedings were opened by the Mayor of Harrogate, Dr. Neville Williams, after which the President, Dr. Tyson, took the chair. Mr. C. Fox Strangways then read a paper on the "Geology of Harrogate in Relation to its Mineral Waters," which was followed by a paper by Dr. Frederick Lever, on "The Harrogate Waters and their Therapeutics." At the dinner on May 16 at the Hôtel Majestic Dr. Edgecombe said that the spas and watering-places of this country owe a debt of gratitude to the British Balneological and Climatolgical Society for the work it has done and is doing in driving home to the minds of the medical men of this country the fact that here in the British Isles we have spas equal in value to any abroad, and that many patients hitherto sent abroad can equally well be treated at a home resort. He said that members of the profession practising at health resorts are under a deep obligation to the Society for its work in helping to raise spa treatment from the slough of empiricism to the domain of rational and scientific therapeutics. On the Sunday the Dean of Ripon preached a sermon in Ripon Cathedral, the subject being "The Pool of Bethesda."

At the last annual meeting of managers of the Metropolitan Asylums District, held on May 23, Mr. J. T. Helby as re-elected chairman and Mr. J. Thornley vice-chairman.

The University of Oxford proposes, on June 24, to confer he honorary degree of D.Sc. upon Dr. Fulgence Raymond, f the Hôpital de la Salpêtrière, Professor in the University of Paris.

Lord Cawdor, Treasurer of the London Homœopathic Hospital, has received the sum of 1,000 guineas for the endowment of a bed in the new extension in memory of Dr. George Napoleon Epps from his daughters and sons; also the transfer of £500 Consols bequeathed to the hospital by the late Mr. George Fielder.

The King has been pleased to give to Marc Armand Ruffer, Esq., C.M.G., M.D., President of the Egyptian Maritime Sanitary and Quarantine Council, his Majesty's authority to accept and wear the Insignia of Commander of the Order of the Crown of Italy, conferred upon him by the King of Italy, in recognition of valuable services rendered by him.

It is proposed to hold an Indian Medical Congress in Bombay during the early part of next year. We learn that a preliminary meeting to discuss the methods and scope of the Congress was held lately at Government House, Bombay, under the presidency of the Governor. A central committee was then formed for the purpose of drawing up a definite programme for the Congress.

The election of members of the Council of the Royal College of Surgeons of England will take place on July 2. There will be four vacancies. Mr. Howard Marsh has withdrawn from the Council before the end of his term of office. Mr. Ward Cousins and Mr. Pearce Gould retire at the conclusion of their full term, and Mr. W. Harrison Cripps retires in his capacity as substitute member since 1905 for the late Sir Alfred Cooper, who was elected in 1900.

The members of the Imperial Yeomanry Hospitals Committee, the staff, and some of its principal subscribers have sent £221 11s. towards the "Hall-Edwards" Fund as a mark of appreciation of the services rendered during the African war by Dr. Hall-Edwards, who has suffered so much as the sult of his $x$-ray investigations. The Imperial Yeomanry School Committee have also forwarded a cheque for £100 towards the fund. This brings the total sum subscribed to £1,956.

The sixty-third anniversary dinner in aid of the funds of the German Hospital, Dalston, was held on May 22 at the Hôtel Métropole. The Lord Mayor, who was accompanied by the Sheriffs, occupied the chair, and, in proposing the toast of "The Hospital," said the institution was doing a great work in the North-East of London, which was otherwise almost destitute of hospital accommodation. It was largely used by English patients from the surrounding districts, and all cases of accident, no matter what the nationality of the injured person might be, were at once admitted. A new convalescent home at Hitchin, the gift of Messrs. Fritz and Hans Koenig, was nearing completion, and would be ready for occupation early in July. A sum of £10,000 had already been received from two warm friends of the hospital—Baron von Schröder and his uncle—towards the endowment of the home, but further help was needed. Donations and subscriptions were announced amounting to upwards of £4,300, including £200 from the German Emperor and £50 from the Emperor of Austria.

The report of the Treasurer (Mr. Cosmo Bonsor) upon the work done during 1907 at Guy's Hospital states that at the close of the year all loans had been discharged and the hospital, chiefly through the testamentary beneficence of Mr. Alfred Beit and Mr. Samuel Lewis, stood free from debt, for which welcome relief the Governors desire to express their warm gratitude. It brings them to the position of having completed and paid for practically all the works included in the appeal of 1901, restated in the supplementary appeal of 1905, and clears the way for a concentration of effort upon the very necessary tasks which remain, the most immediate of which are the augmentation of regular income and the provision of those further special works enumerated in the previous report, namely, a new outpatient department, separate children's wards, and the rebuilding of clinical house. The first of these works has already been proceeded with.

The annual dinner of the Association of Scottish Medical Diplomates was held at the Waldorf Hotel, London, on Tuesday, May 26. Dr. David Walsh, the President of the Association, was in the chair, and a very pleasant evening was spent. Among those who spoke after dinner were Dr. H. MacNaughton Jones, Dr. J. G. Fitzgerald, and Dr. McManus, one of the direct representatives upon the General Medical Council. The Association has now been in existence for four years, and, with increased support from the many holders of Scottish medical and surgical diplomas resident in the South, its useful work should become much extended. The President alluded to the fact that medical men holding the Scottish conjoint diplomas are at a disadvantage compared with those holding the double qualification of the English Colleges in not possessing a member's diploma of the College of Surgeons, and he expressed the hope that the Edinburgh College would extend this privilege to its licentiates.

### The King's Hospital Fund.

### MUNIFICENT GIFT BY LORD MOUNT STEPHEN.

The Prince of Wales, as President of King Edward's Hospital Fund for London, has received from Lord Mount Stephen 5,000 shares of the Great Northern Railway Company of the United States, yielding an annual income of roughly £7,000, as an addition to the invested capital of the Hospital Fund. This munificent gift represents at the present market value of the shares a capital sum of about £130,000, the nominal value being £100,000. Lord Mount Stephen's previous gifts to the Fund were £200,000 in 1902 and £200,000 in 1905.

The Fund, it will be remembered, was founded in 1897 by His Majesty, then Prince of Wales, to commemorate the sixtieth anniversary of the accession of Queen Victoria. An annual sum of £100,000 to £150,000 was then suggested as necessary to place the hospitals on a sound financial basis. But, as the Prince of Wales pointed out in his speech at the annual meeting in 1905, "since then the population of London has grown, its building area has been enlarged, hospital requirements have increased. To meet these growing demands I am convinced that our aim must be to provide from the King's Fund an income from all sources which will permit of an annual distribution among the London hospitals of at least £150,000 a year." The amount distributed to the hospitals of London by the Fund last year was £120,000, which exceeded the income from investments by £70,000. Lord Mount Stephen's munificent gift raises the total income of the Fund from investments to nearly £60,000 a year, and he hopes to see the income from investments increased to £75,000 a year.

